# The utilization of simulated patients for teaching and learning in the pharmacy curriculum: exploring pharmacy students’ and recent alumni’s perceptions using mixed-methods approach

**DOI:** 10.1186/s12909-021-02977-1

**Published:** 2021-11-06

**Authors:** Hager ElGeed, Maguy Saffouh El Hajj, Raja Ali, Ahmed Awaisu

**Affiliations:** Department of Clinical Pharmacy and Practice, College of Pharmacy, QU Health, Qatar University, P.O. Box 2713, Doha, Qatar

**Keywords:** Competency-based curriculum, Curriculum, Performance-based assessment, Pharmacy education, Simulated patients, Students’ perception

## Abstract

**Background:**

The use of simulated patients (SPs) is considered a significant resource for teaching and assessing clinical and communication skills in health professional education. We conducted this study to explore pharmacy students’ perspective towards the utilization of SPs in Qatar and to identify areas that require improvement.

**Methods:**

An explanatory sequential mixed-methods design was used among students and recent graduates of the College of Pharmacy at Qatar University (QU-CPH). First, their perspectives toward the current utilization of SPs at QU-CPH was explored using a quantitative cross-sectional study design. Following this, we conducted six focus group discussions based on the analysis of the questionnaire results. The findings of the two phases were interpreted through integration of the quantitative and qualitative phases.

**Results:**

The majority of the participants (> 90%) reported that interactions with SPs are important in building good communication and counseling skills during professional skills course activities. Similarly, most of the respondents (80%) indicated that interactions with SPs prepared them to apply the clinical skills gained during professional skills and patient assessment sessions in real-life. In addition, they reported that interactions with SPs during competency-based assessments were good experiences. The participants disagreed with the notion that interaction with SPs of opposite gender was uncomfortable for them. Themes identified from the focus groups include: interactions with trained SPs compared to faculty SPs, standardization and consistency of SPs’ roles, communication and language barriers, simulations of real-life case scenarios, SPs’ competence and preparedness, psychological impact associated with interaction with SPs, proposed strategies for improving the SP program. Identified areas for improvement include the need for strengthening the SP training and orientation program as well as the SP selection criteria.

**Conclusion:**

This study showed a positive impact of the utilization of SPs in this pharmacy curriculum as perceived by students and alumni. However, the SP program needs to be optimized in terms of the training and orientation of SPs.

## Background

Healthcare professional education and training, including pharmacy education, requires the use of a variety of instructional and assessment strategies to help students develop several skills, such as collecting patient data and history-taking, performing physical examinations, creating an appropriate care plan and designing a monitoring plan [1]. In pharmacy education, clinical and communication skills are among the major skills and competencies that students should gain during their undergraduate education. These skills can be taught and assessed in multiple ways including through performance-based assessments that involve the use of simulated patients (SPs) [1, 2]. An SP is defined as an actor/actress who is trained to represent a patient during a clinical encounter with a healthcare provider or a healthcare professional student [3]. The use of SPs is considered to be a significant resource for teaching and assessing clinical skills, including physical examination, communication, data gathering, patient counseling, and education [4, 5]. The published literature has documented the benefits of the utilization of SPs compared to the utilization of faculty member or peer student actors portraying the role of patients in medical education [4, 6]. Studies have also found that incorporating SPs in courses where communication is a major learning outcome resulted in better student performance in different health-related disciplines including nursing, medicine, pharmacy, and physical and occupational therapy [5, 7–11]. It is believed that the utilization of SP method provide pharmacy educators with a tool for implementing communication skills in the practice of pharmacy and will serve as a basis for implementing communication skills development programs; and this will help students to excel in problem-based scenarios and real life simulation [12]. SPs serve as a bedrock of simulation-based education. The commonest principal learning theories that are associated with and contribute to the design and practice of simulation-based learning include the behavioral, constructivist, and social cognitive conceptual frameworks [13, 14]. These theoretical concepts consider the social character of simulation and allow for improved matching of simulation realism with desired outcomes when designing and conducting scenarios [14]. Therefore, curricular design and utilization of SPs in simulation-based learning should consider these conceptual frameworks.

As the use of SPs is believed to improve patient-provider communication and simulate real-life situations [4, 6, 15–17], the College of Pharmacy at Qatar University (QU-CPH) initiated an SP program in 2015. QU-CPH was established in 2007, and it is currently the only pharmacy school in Qatar. It is a Canadian-accredited pharmacy program that follows a world-class patient care-oriented curriculum [18]. Effective communication is a core skill for healthcare professionals that enables them to be competent healthcare providers who can contribute to the achievement of healthcare outcomes, and it is a skill that helps them build effective interprofessional relationships [19].

Prior to the introduction of the SP program at QU-CPH in 2015, the college utilized multiple teaching and assessment approaches including the involvement of employees (administrative staff and faculty members) playing patient roles to optimize the communication skills of pharmacy students. This approach was labor intensive and became more difficult when the number of students admitted to the program increased, the curriculum progressively advanced, and the number of performance-based assessments increased accordingly. In order to account for the new demands, QU-CPH established the SP program to improve the integration and application of knowledge and students’ learning experiences. Courses that involve SPs are typically professional skills and patient assessment courses and the integrated case-based learning course series. Similarly, assessment types in these courses that involve the use of SPs include Structured Multi Skill Assessments (SMSAs) and Objective Structured Clinical Examinations (OSCEs), in addition to physical assessment practical examinations. SMSA is a modified version of the OSCE and a performance-based assessment method invented at the QU CPH that addresses contextual and cultural considerations when used in undergraduate pharmacy curricula [20]. It was created to provide an affordable performance-based assessment model for educators working within resource-constrained settings [20].

A few studies have examined the perspectives of healthcare professional students regarding the utilization of SPs in pharmacy education and training [8, 16, 19] while other studies have focused on medical and nursing students’ perceptions [7, 21] and the perceptions of students from other specialties such as speech therapy and occupational therapy [11, 22]. From the introduction of the SP program at QU-CPH to date, no study has been conducted to explore students’ perceptions of using SPs for learning and assessment. As we utilize SPs extensively, it is recommended that we work on developing clear guidelines to improve the SP program [23]. Moreover, understanding the perspectives of pharmacy students and recent graduates will enrich the continuous quality improvement process of the program and will benefit other pharmacy colleges globally. This study primarily aimed to explore pharmacy students’ and recent graduates’ perceptions of the use of SPs in pharmacy education and how the utilization of SPs influenced their professional education. The secondary objective of the study was to identify areas that may require improvement to optimize the utilization of SPs in teaching-learning and assessment in the undergraduate pharmacy curriculum.

## Methods

### Study design and setting

This study was conducted at QU-CPH in Qatar, the only pharmacy college in the state of Qatar that offers a BSc, PharmD, MSc and PhD degrees in pharmacy. The college, which accepted its first batch of students in 2007, is accredited by the Canadian Council for Accreditation of Pharmacy Programs (CCAP). The program largely follows a competency-based curriculum and provides BSc and PharmD students with opportunities to learn clinical and professional skills through simulation-based learning. The BSc program currently admits about 30–40 students annually.

An explanatory sequential mixed-methods design was used in the study [24–27]. The students’ and recent graduates’ perspectives toward the current utilization of SPs at QU-CPH were explored using a quantitative questionnaire-based study design (Phase 1). In order to obtain in-depth perspectives of representative students, we conducted a series of focus group discussions (FGDs) based on the analysis of the questionnaire results (Phase 2). Finally, we interpreted the findings of the two phases through integration of the quantitative and qualitative findings.

### Study population

All current BSc pharmacy students from professional year 2 to professional year 4 and alumni who graduated in spring 2018 and spring 2019 (*n* = 133) from QU-CPH were invited to participate in the study. We excluded pharmacy students in their first professional year because they had very limited or no interactions with SPs and excluded alumni who graduated before the spring semester of 2018 to minimize any potential for recall bias.

### Sample size and sampling

Based on the identified population who met the inclusion criteria (*n* = 133), the minimum sample size of 99 was calculated with a 95% confidence interval and 5% margin of error using the Raosoft^®^ online sample size calculator to participate in the first phase of the study. However, because the population was small, we used a whole population sampling approach in which we invited all members of the study population to participate.

### Phase 1: quantitative questionnaire-based study

An online survey was conducted using a piloted questionnaire. An initial invitation containing the link to the online questionnaire was sent to the study sample and was followed by follow-up reminders on a weekly basis for 6 weeks. The initial email identified the purpose of the study, consent to participate and the link to the survey.

#### Questionnaire development and validation

The study investigators developed the questionnaire based on a review of the literature and the pedagogical questions of the investigation [5, 10, 17, 19, 21, 28, 29]. The initially developed questionnaire was revised by the study investigators and was later revised by faculty members with expertise in pharmacy education and questionnaire development. Several modifications were made to the first draft of the questionnaire through interactions between reviewers and the investigators.

The questionnaire was piloted using some former graduates of the college who were not eligible to participate in the study. The final version of the questionnaire was constructed based on an iterative process and consensus among three of the investigators. The questionnaire comprised 29 items and three demographic questions. The questionnaire was divided into three sections to assess perceptions on the following: [1] the utilization of SPs in professional skills courses (10 items), [2] the utilization of SPs in physical assessment courses (7 items), and [3] the utilization of SPs during performance-based and practical assessments (11 items). All items were measured on a Likert scale ranging from “strongly agree” to “strongly disagree” with a last option of “cannot recall”. The final version of the questionnaire was distributed to the participants using the SurveyMonkey^®^ online software (SurveyMonkey Inc., San Mateo, California, USA). The link was distributed via personal e-mails to the study population. The language of the questionnaire was English as it is the official language of instruction at QU-CPH. A copy of the questionnaire can be obtained through the corresponding author.

#### Survey administration and data collection

The questionnaire was completely anonymous, and there were no means to link the respondents with their respective responses. The questionnaire URL link was open from 23 July to 6 September 2020. Five reminders were sent to participants during the study period to increase the response rate. The responses received were reviewed after closure of the online link to the survey, and respondents who filled in only Section A (demographics) either completely or partially were excluded from the analysis because such responses will not add any value to the study.

### Phase 2: qualitative focus group discussions

Students and recent graduates who participated in the quantitative phase of the study were invited to participate in the FGDs [30] in order to obtain an in-depth understanding of their perspectives on SP utilization in the pharmacy curriculum and to know more about their recommendations to improve the program. FGDs were led by one of the research team members who has experience in conducting FGDs.

#### Focus group guide and setting

The interviews were based on a pre-determined interview guide that was prepared and reviewed by the research team. Interviews took place between 21 September, 2020 to 8 October, 2020. Each focus group session was composed of 4–7 participants and lasted for approximately 60 min. The concept of theoretical saturation was followed. Following the sixth focus group, no new ideas emerged, and saturation was judged to be achieved by the investigators.

Thirty-three current female students and recent graduates participated in the six FGDs (19 current BSc students and 14 graduate). Each FGD session comprised a mix of current students and recent graduates. In addition, the participants were from different countries, and different professional years or years of graduation. Some of the recent graduates were employed and practicing as pharmacists in different healthcare settings in Qatar.

#### Qualitative data collection and analysis

The interview guide for the FGDs was developed based on the literature review used in Phase 1 and the study objectives. The guide was developed and validated by the study investigators. All FGDs were conducted virtually through the Microsoft Teams^®^ application due to the current COVID-19 pandemic. All FGDs were audiotaped using the recording feature in the Microsoft Teams^®^ platform upon obtaining consent for recording from all participants. The FGDs were transcribed verbatim. A thematic data analysis approach was used; a qualitative analysis framework was generated that allowed for structuring, labeling and defining data. Each phrase was coded with a code that reflects the meaning in the text. Similar phrases, paragraphs, and ideas were sorted together under the same code, and the codes were used to generate themes.

### Data analysis

For the quantitative phase, the data were extracted from SurveyMonkey^®^ to IBM Statistical Package for Social Sciences (IBM SPSS^®^ Statistics for Windows, version 26.0; IBM Corp, Armonk, NY, USA). Descriptive analysis was primarily used for the quantitative part of the study. The data of the categorical variables are presented as frequencies and percentages. For the qualitative phase, thematic content analysis was used to structure the information derived from the FGDs, as described above. Coding was manually performed by the study investigators. The Standards for Reporting Qualitative Research (SRQR) tool was utilized to improve the quality of the reporting of the qualitative component of the study [31].

### Ethical considerations

The study protocol, the informed consent forms, the questionnaire, and the FGD guide were reviewed and approved by the Qatar University Institutional Review Board (approval number QU-IRB 1331-EA/20). The privacy and confidentiality of the study participants were protected at all times throughout the study. Furthermore, the questionnaire was anonymous, and participation was voluntary. The data collected would not be shared with anyone outside the research team and would be deleted after 5 years as per the regulations of the ethics committee.

## Results

### Quantitative phase

#### Participants’ demographic characteristics

Of the 133 eligible students and recent alumni who were invited to participate in the study, 103 completed the online questionnaire (response rate, 77.4%). All participants were female, because the QU CPH was a gender-segregated college with only female students at the time of the survey. Approximately 39 (37.8%) were recent graduates while the rest were students in professional years 2–4. As shown in Table 1, we had similar response rates from the three professional years. One-third (33%) of the participants were from Egypt, followed by Syria (11.7%) and Palestine (10.7%). The other participants were from countries such as Sudan, Tunisia, Pakistan, and Algeria. More details about the demographics of the participants are presented in Table 1.

**Table 1:** Demographic characteristics of pharmacy students and recent graduates (*n* = 103)

Characteristic	n (%)
**Professional year**	
Professional year 2	21 (20.4)
Professional year 3	22 (21.4)
Professional year 4	21 (20.4)
Graduated with a BSc (2018)	19 (18.4)
Graduated with a BSc (2019)	20 (19.4)
**Nationality**	
Qatari	6 (5.8)
Egyptian	34 (33)
Palestinian	11 (10.7)
Syrian	12 (11.7)
Others	40 (38.8)
**Gender**	
Female	103 (100.0)

#### Perceptions of pharmacy students/graduates regarding the use of simulated patients in professional skills course activities

Most participants disagreed with the notion that interacting with a student colleague was preferred over an SP (53.4% vs. 25.2%) while 21.4% were neutral. Similar responses were seen when participants were asked if they preferred interacting with an instructor to interacting with an SP (Table 2). Most participants (92.3%) found that interactions with SPs are important in building good communication skills during professional skills course activities. Similarly, more than 90% of the participants found the interactions with SPs helpful for building their education and counseling skills during professional skills course activities. Approximately 80% of the respondents indicated that interactions with SPs prepared them to apply the clinical skills gained during professional skills sessions in real life. More details on the perceptions of pharmacy students/graduates regarding the use of simulated patients in professional skills course activities are summarized in Table 2.

**Table 2:** Perception of pharmacy student/graduate about the use of simulated patients in Professional Skills course activities (n = 103)

Learning and assessment activity item	Responses, n (%)					
	***Strongly agree***	***Agree***	***Neutral***	***Disagree***	***Strongly disagree***	***Cannot recall***
Interacting with a student colleague(s) is/was preferred over interacting with SPs during Professional Skills course activities.	7 (6.8)	19 (18.4)	22 (21.4)	35 (34)	20 (19.4)	0 (0)
Interacting with a course instructor(s) is/was preferred over interacting with SPs during Professional Skills course activities.	13 (12.6)	19 (18.4)	19 (18.4)	38 (36.9)	14 (13.6)	0 (0)
Interacting with SPs is/was important for me to build good communication skills during Professional Skills course activities.	49 (47.6)	46 (44.7)	4 (3.9)	4 (3.9)	0 (0)	0 (0)
Interacting with SPs makes/made me LESS confident in data gathering process during Professional Skills course activities	1 (1)	15 (14.6)	15 (14.6)	43 (41.7)	28 (27.2)	1 (1)
Interacting with SPs is/was helpful for me to be systematic in data gathering process during Professional Skills course activities.	19 (18.4)	54 (52.4)	24 (23.3)	6 (5.8)	0 (0)	0 (0)
Interacting with SPs makes/made me LESS confident in providing patient counseling and education during Professional Skills course activities.	2 (1.9)	15 (14.6)	9 (8.7)	47 (45.6)	30 (29.1)	0 (0)
Interacting with SPs is/was helpful in building my patient education and counseling skills during Professional Skills course activities.	38 (36.9)	56 (54.4)	6 (5.8)	3 (2.9)	0 (0)	0 (0)
Interacting with SPs is/was NOT reflective of real-life situations during Professional Skills course activities.	3 (2.9)	18 (17.5)	20 (19.4)	44 (42.7)	18 (17.5)	0 (0)
Interacting with SPs is/was useful for me to apply my clinical theoretical knowledge during Professional Skills course activities.	25 (24.3)	63 (61.2)	11 (10.7)	4 (3.9)	0 (0)	0 (0)
^a^Interacting with SPs has prepared me to apply clinical skills and knowledge gained during Professional Skills in real life.	29 (28.2)	53 (51.5)	14 (13.6)	4 (3.9)	0 (0)	2 (1.9)

^a^ 1 missing data

#### Perceptions of students/graduates regarding the use of simulated patients in patient assessment course activities

Most students and recent graduates indicated that interacting with an instructor is less preferred than interacting with SPs (32% versus 51.5%). Most participants found that conducting patient assessment using SPs was helpful for them to observe patients’ verbal responses (78.6%). In addition, 82.5% of the participants found that interacting with SPs was helpful for them to apply theoretical knowledge of physical examinations during patient assessment course activities. Further details are presented in Table 3.

**Table 3:** Perception of student/graduate about the use of simulated patients in Patient Assessment course activities (n = 103)

Learning and assessment activity item	Responses, n (%)					
	***Strongly agree***	***Agree***	***Neutral***	***Disagree***	***Strongly disagree***	***Cannot recall***
Interacting with a student colleague(s) is/was preferred over interacting with SPs during Patient Assessment course activities.	2 (1.9)	22 (21.4)	26 (25.2)	38 (36.9)	14 (13.6)	1 (1)
Interacting with a course instructor(s) is/was preferred over interacting with SPs during Patient Assessment course activities.	9 (8.7)	24 (23.3)	17 (16.5)	38 (36.9)	15 (14.6)	0 (0)
Conducting patient assessment on a mannequin is/was better than using SPs during Patient Assessment course activities.	9 (8.7)	20 (19.4)	13 (12.6)	41 (39.8)	16 (15.5)	4 (3.9)
Conducting patient assessment using SPs helps(ed) me to observe patient’s verbal responses.	24 (23.3)	57 (55.3)	11 (10.7)	6 (5.8)	1 (1)	4 (3.9)
Conducting patient assessment using SPs helps(ed) me to observe patients’ non-verbal attitudes (e.g. reactions and feelings.)	29 (28.2)	51 (49.5)	9 (8.7)	9 (8.7)	1 (1)	4 (3.9)
Interacting with SPs helps(ed) me to apply my theoretical knowledge of physical examination during Patient Assessment course activities.	26 (25.2)	59 (57.3)	10 (9.7)	3 (2.9)	0 (0)	5 (4.9)
Interacting with SPs improves/improved my confidence in conducting the patient assessment during Patient Assessment course activities.	24 (23.3)	53 (51.5)	15 (14.6)	7 (6.8)	0 (0)	4 (3.9)

#### Perceptions of students/graduates regarding the use of simulated patients in practical examinations (SMSA/OSCE and practical patient assessment examinations)

Approximately 71.6% of the participants reported that the interactions with SPs during the SMSA and OSCE were good experiences. This was also the case regarding the use of SPs in practical patient assessment examinations, as 79.4% found it to be a good experience. Fifty-one percent of the participants agreed that the SPs were well trained on and familiar with the cases they simulated during practical examinations while more than 14% disagreed with this. When the participants were asked if the SPs remained in their roles throughout the duration of the interaction, more than 65% agreed while 13.8% disagreed and 20.6% were neutral. Most participants (56.8%) agreed that the SPs’ demographics were generally compatible with the cases presented in practical examinations while 19.6% were neutral and 15.7% disagreed with this. For more details, refer to Table 4.

**Table 4:** Perception of student/graduate about the use of simulated patients in practical examinations and performance-based assessments (OSCE/SMSA) (*n* = 102)

Learning and assessment activity item	Responses, n (%)					
	***Strongly agree***	***Agree***	***Neutral***	***Disagree***	***Strongly disagree***	***Cannot recall***
Interacting with SPs during SMSAs and/or OSCEs is/was a good experience.	17 (16.7)	56 (54.9)	18 (17.6)	9 (8.8)	2 (2)	0 (0)
Interacting with SPs during Patient Assessment practical examinations is/was a good experience.	25 (24.5)	56 (54.9)	14 (13.7)	4 (3.9)	2 (2)	1 (1)
^a^Utilization of SPs during practical examinations is/was effective in assessing my clinical knowledge.	24 (23.8)	53 (52.5)	17 (16.8)	5 (5)	2 (2)	0 (0)
Utilization of SPs during practical examinations is/was effective in assessing my patient education and counseling skills.	24 (23.5)	60 (58.8)	14 (13.7)	1 (1)	3 (2.9)	0 (0)
Overall, the SPs are/were well trained and oriented about the cases they simulate during the practical examinations.	11 (10.8)	41 (40.2)	35 (34.3)	12 (11.8)	3 (2.9)	0 (0)
Overall, the SPs always remain(ed) in their role (i.e. they stick to the scenario) throughout the duration of the interaction.	13 (12.7)	54 (52.9)	21 (20.6)	12 (11.8)	2 (2)	0 (0)
I find/found practical examinations without SPs more effective than having the practical examinations using SPs.	2 (2)	12 (11.8)	19 (18.6)	49 (48)	16 (15.7)	4 (3.9)
Interacting with SPs during the practical examinations is/was more stressful than having a member of the college acting out the scenario.	7 (6.9)	18 (17.6)	15 (14.7)	44 (43.1)	16 (15.7)	2 (2)
Interacting with SPs during the practical examinations induces(ed) more anxiety than interacting with a member of the college acting out the scenario.	8 (7.8)	17 (16.7)	15 (14.7)	44 (43.1)	17 (16.7)	1 (1)
I find/found the SPs demographics (e.g. age, gender, ethnicity…etc) generally compatible with the case scenarios they are/were acting.	8 (7.8)	50 (49)	20 (19.6)	14 (13.7)	2 (2)	8 (7.8)
I prefer(ed) interacting directly with the SPs without the assessor being in the room during the practical examinations.	32 (31.4)	27 (26.5)	21 (20.6)	11 (10.7)	7 (6.9)	4 (3.9)

^a^1 missing data

#### Overall perceptions about simulated patients’ gender

**Fig. 1:**
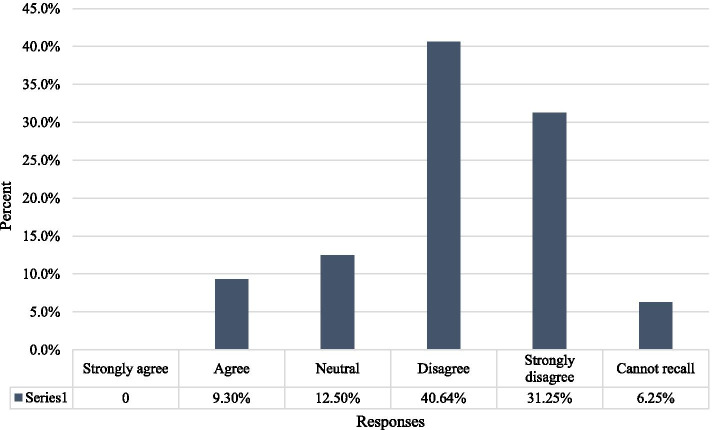
Overall perception about simulated patients’ gender (*n* = 101)

Finally, the participants were asked if they found the interaction with an SP from the opposite gender (male SPs) to be uncomfortable for them. More than 71% of the participants disagreed with this notion (Fig. 1).

### Qualitative phase

#### Themes generated from the focus groups

Seven major themes emerged from the FGDs conducted as part of this study, and these themes include the following: interactions with trained SPs compared to faculty SPs, standardization and consistency of SPs’ roles, communication and language barriers, simulations of real-life case scenarios, SPs’ competence and preparedness, the psychological impact associated with interaction with SPs, and proposed strategies for improving the SP program. The major themes and categories are summarized in Table 5.

**Table 5:** Themes and Subthemes Generated from the Qualitative Analysis

***Theme 1: Interactions with real SPs compared to faculty SPs*** Preference for interaction with real/trained SPs over faculty SPs Preference for faculty SPs over real SPs No preference over who acts (real SPs in comparison with trained SPs)	
***Theme 2: Standardization and consistency of SPs’ role*** Lack of standardization of SPs’ role and inconsistency in information provision Variability in SPs’ acting skills and performance Variability in SPs’ mannerism	
***Theme 3: Communication, language and other barriers*** Issues with SPs’ verbal communication skills Issues with SPs’ non-verbal communication skills SPs’ not initiating the interaction with students causing confusion	
***Theme 4: Simulation of real-life case scenarios*** Interaction with SPs is realistic and simulates real-life experiences Diversity of the SP pool and their characteristics Unrealistic experience in terms of language, SPs’ literacy, number of diseases and duration of interaction. Demographics of some SPs not fitting case scenario Need to select lay people to be SPs (without medical knowledge)	
***Theme 5: SPs’ competence and preparedness*** Incompetent SPs and lack of preparedness. SPs poor performance and its impact on students’ performance during SMSAs. Choice of SPs with health professional background	
***Theme 6: Psychological impact caused by SPs and performance-based assessment*** Poor SPs’ performance and lack preparedness as a source of stress and anxiety for students Causes of anxiety unrelated to SPs’ performance Impact of students’ anxiety on the interaction	
***Theme 7: SP Program Improvement Strategy*** Recommendations for determining SP selection criteria Recommendations for strengthening SP training Recommendations for overcoming problems with simulation and standardization	

##### Theme 1: interactions with real SPs compared to faculty SPs

This was a predominant theme commonly discussed during the focus groups (FGs) in which the participants expressed their opinions regarding their interactions with real SPs compared to faculty SPs and their preferences in this regard. The preference for interactions with real SPs over faculty members was a commonly expressed opinion among the participants in this study. These participants indicated that they were more comfortable and less anxious during their interactions with real SPs because they are strangers to them; therefore, the participants can treat the SPs as real patients without worrying about being judged by them. Many participants also indicated that interaction with SPs felt more realistic than interacting with faculty members. In addition, many participants found interacting with faculty SPs to be more stressful due to their sense of inferiority in knowledge and experience compared to their faculty members, in addition to their fear of making mistakes in their presence.

*“…but of course, for SPs, as I said, you don’t want to speak to someone you already know or see usually. If I see a faculty member, I get more stressed because I will know that they know better than me and they will be laughing inside if I say something wrong”* (FG4, SA)

*“They can get you into the mood of wanting to provide care for the patient, so it is more realistic; it gives you different experiences from when the SP is a professor because we had instances when the professor was acting out a patient.”* (FG2, ST)

However, another group of the participants indicated their preference for having faculty members or students as SPs at professional skills stations. These individuals felt that faculty members were able to provide more comprehensive feedback to students due to their advanced knowledge and that they tended to cooperate with students better. Conversely, some of the participants indicated that they had no preference for real SPs or faculty member SPs.

*“…but I think the faculty, they have, I don't know… like… I think I am more comfortable with them and their faces, like I did not stress about talking to Dr. ‘X’ or whatever faculty I was going to have; I think I would be more comfortable with them than an SP because some SPs are tall, like really new to us, and I have never had them before in any exam…”* (FG5, DM)

Overall, the participants’ experiences with real SPs varied between positive and negative experiences.

##### Theme 2: standardization and consistency of SPs’ roles

The lack of standardization in SPs’ acting of scenarios and inconsistency in the information provided to students was a recurrent theme in the focus groups conducted with CPH students and alumni. This was particularly evident at parallel stations where two SPs played the same role. The differences noticed between SPs were in terms of the acting skills, SP mannerisms and the information provided. Participants reported that the variability in SP acting skills affected students’ performance in some cases as it limited their ability to identify and address the underlying problem.

*“I remember in one of the SMSA, there was a Parkinson’s station. Once, half of our group couldn’t even tell it was Parkinson’s, while the other half said oh, it was so obvious, it was an easy station. Literally, some groups suffered, and some found it easy because there was a very obvious tremor. In our group, the SP had a very low voice, so we couldn’t even tell what the problem was and we were meant to know that there was a tremor, but she didn’t even act it our properly.”* (FG2, ST)

The participants also reported that the SPs acting in the same case at parallel stations could sometimes provide different information or might miss giving the students answers to important questions that, as a result, affect the students’ performance. It was also suggested that sometimes the same SP might provide different answers to different students and highlighted the importance of having a standard answer for each question.

*“…there was, like, an imbalance between the information we were getting from SPs in different stations; for example, my friend, she got the information and she did the counselling well but then for me… I… she either didn't tell me or she did not, like, she gave me like the wrong information so I could not counsel her on that. So I missed points there, and I think there was an imbalance in this regard.”* (FG1, HS)

The participants also highlighted the variability in SPs’ personalities and attitudes and its impact on their performance as they indicated that some SPs are comforting, others are difficult to handle and some are willing to speak while others tend to be reluctant to answer questions and talk.

*“Some SPs are really open and helpful, while for others, we need to pull the information from them.”* (FG6, MA)

##### Theme 3: communication, language and other barriers

Communication problems with SPs were frequently mentioned by the participants during the FGs. The most commonly reported issues were related to information delivery to students as it was suggested that some SPs miss giving important information while others provide all information without allowing students to ask. SPs’ pace of speech and accents were also highlighted as factors that could affect the interaction.

*“…for example, last year we had a hypertension case and I asked her if she was taking any other medication and then she was like: ‘No, I don't take any other medication’, so Dr. ‘Y’ gave her the medication because she was supposed to tell me that she was taking it. So that's it; sometimes they forget…”* (FG3, AA)

*“Sometimes their accent is bad, especially when they say the name of meds, so I asked her to repeat multiple times for me to understand what she says”* (FG6, RT).

The participants also indicated that SPs’ body language could sometimes confuse the students, especially when it is not consistent with the emotions they express during the interaction.

##### Theme 4: simulation of real-life case scenarios

The interaction with SPs during professional skills courses was generally found to be realistic and simulated real-life experiences. During the FGDs, the participants talked about the diversity of the SPs included in the SP program in terms of their nationalities, cultures, accents, healthcare beliefs and practices. This diversity was found to be beneficial in building cultural competence among the students.

*“Ok, so most of the times that I had encounter with an SP, like, the performance was always good and some of them actually acted out the case, like NI was saying. So that helps you concentrate; even if it's an exam, it makes you feel like it's real-life situation”.* (FG1, MM)

However, some of the participants suggested that the SPs in this program were not representative of the real patient population due to the absence of some special populations including pediatrics and individuals with special needs. Furthermore, other participants found the experience to be unrealistic because all patients were educated and had multiple illnesses, and the participants recommended including SPs without a medical background. Likewise, the duration of the interaction did not reflect reality to some participants as in real life, there are no time limits. The lack of compatibility of the SPs’ demographics with that of the case scenario was another issue that limits the realistic aspect of the interaction.

*“I had one encounter during my SMSA where the person sitting in front of me was a woman but her name was Yasser (male’s name). And during the whole session I didn't know if she was a man or a woman and I ended up asking her if she was pregnant and* [32] *that she was actually playing the role of a male patient…”* (FG3, MR)

##### Theme 5: SPs’ competence and preparedness

Some participants felt that some of the SPs were incompetent and were not prepared to act scenarios at professional skills stations as these individuals were often struggling to find read answers from written materials, provide wrong or misleading information and possess a lack of confidence and need for confirmation from assessors.

*“Sometimes when you ask them a question that they don’t know the answer to, they start looking at the doctor who is with us. If the doctor asks them to move on, they will move or come up with an answer that will totally ruin the whole case.”* (FG4, AS)

Some of the issues brought up by some participants were that some SPs lack self-confidence when responding or wait for confirmation from professors or assessors to answer the students. As per the participants, this was difficult for them as students.

“… *and confidence also… because I remember an SP once in the language barrier station - I was trying very hard, like I tried all the techniques, and the SP was not confident at all of whether or not they should interact and they would always look at the professor to figure out "Should I continue, should I sit?" Like, I can see in their eyes they're looking to get an “Ok”, do you know what I mean? It was so very distracting and made it very slow…”* (FG2 A2)

Choosing competent SPs with backgrounds in healthcare, including CPH graduates, was recommended during the FGDs, especially for individuals playing the role of physicians.

*“One more thing. The SPs who have some medical background—some of them are nurses, some of them are students in CPH—they know what they are talking about. They don’t change their information, and they say it in the way I want to hear it…”* (FG2, LS)

##### Theme 6: psychological impact caused by SPs and performance-based assessment

Several factors were reported to be the source of students’ stress and anxiety during SMSAs, including poor SP performance and lack of preparedness, unclear expectations from students, the simulation and time restrictions. It was also suggested that having professors as SPs increases students’ stress levels. Students’ anxiety affects their performance and the quality of the interaction.

*“… It's kind of stressful because you don't know what they are expecting you to ask. At the same time, if you ask a question, sometimes they will never answer what you would like to hear.”* (FG4, AS).

##### Theme 7: SP program improvement strategy

The participants in the FGDs provided different recommendations to help improve the SP program by improving the SP selection criteria and training and overcoming simulation- and standardization-related problems. Participants recommended expanding the SP selection criteria to include individuals from different nationalities who speak different languages with special emphasis on the Arabic language. Some highlighted the importance of SPs’ personalities during the selection, and it was recommended to include individuals who pay attention during sessions and give feedback. It was also advised to include real patients as SPs and to ensure the compatibility of the SPs’ demographics with the case scenario. The participants also suggested considering students’ feedback on SPs’ performance.

*“…So, I believe SPs speaking in Arabic or another language that requires translation will give us much more experience. Having that interaction is good, like with a Hindi SP or any language, and of course in Arabic, not only English.”* (FG2, MH)

The need for SP training and orientation was a predominant subtheme that appeared frequently in all the FGDs. Generally, there was a belief that the orientation and training sessions currently provided were not adequate, and participants sensed the need for improving SP training by developing training programs for SPs supervised by faculty members with mock tests before assessments. It was also recommended that comprehensive orientation sessions for SPs with an emphasis on the key points SPs should never forget should be developed.

*“…So, I would really recommend that before they take the exam, one of the doctors sits with them and pretends he is a student and they practice the whole counselling and see if they know how to respond or not...”* (FG5, KA)

It was recommended that a registry for regular SPs be developed and that SPs who are confident and have good communication skills be selected. Participants also suggested involving SPs in regular practical sessions and not only in assessments. Seeking assessors’ feedback on SP performance was also deemed to be important in SP selection, and it was recommended that surveys be developed for this purpose. To improve the standardization, the participants suggested having one SP per case, rehearsal sessions between two SPs who would play the same scenario and having role-playing sessions with faculty members. Ensuring similar demographics between SPs playing the same role and recording the interactions were also recommended. To ensure the consistency in the information provided by SPs, participants recommended distributing written scenarios or checklists with answers for questions.

*“All what you can do is train them, pick those who speak clearly and act clearly and confidently. Sometimes the SP is really shy. We had an SP who was so shy that I was scared to ask her to speak.”* (FG4, SA)

*“...or make like a survey after the SP finishes, a confidential survey to say if this SP is always doing a very good job or this SP needs more training. Something like that because we never gave our opinion about the SPs; all of them just came randomly.”* (FG2, ST)

To enhance the simulation of the cases, it was recommended that the time dedicated for each station be increased, that real patients be recruited as SPs and that site visits for SPs be offered during which they can meet real patients. Participants also highlighted the need for introducing SPs to ideal interactions in preparation for their interactions with SPs in the future.

## Discussion

The findings showed an overall satisfaction of the participants with the utilization of SPs in different professional skills-based activities. This was demonstrated in multiple areas of the survey part of the study. For example, most participants preferred interacting with an SP to a colleague or a faculty member when learning or undertaking assessments in professional skills or patient assessment. In addition, most participants acknowledged that interacting with SPs helped them build counseling and education skills. In some previous studies, the involvement of SPs in undergraduate pharmacy education and other professional specialties had a positive impact on the overall performance of the students, especially in activities involving communication [16, 28]. Therefore, we believe that our findings are consistent with those of previous studies. However, it is noteworthy that only a few studies have investigated the perception of SPs by students, especially within pharmacy education.

Similarly, most participants valued the role of SPs in improving their skills in patient assessment courses, which involves learning skills for history-taking, interviewing and the physical examination of different body systems. A previous study assessed the use of SPs in patient assessment courses and found no difference between the use of SPs or a mannequin in performing physical examinations [33]. Another study conducted earlier among pharmacists generated a mixed list of advantages and disadvantages of the utilization of SPs in patient assessment education [34]. In the current study, we found that the use of SPs in patient assessment was rated highly in the quantitative part and during the FGDs. This may be attributed to the fact that in patient assessment, verbal communication with SPs is limited compared to other professional skills courses that require extensive interaction with the patient during consultation, data gathering, patient education and counseling. Therefore, there was a lower probability of standardization deficiencies and misleading information provided by the SPs during the interaction in patient assessment course activities.

The overall perception of the participants in relation to the utilization of SPs in assessments was also mostly positive. Most participants agreed that the utilization of SPs in assessments was a good experience and helpful for them to assess their knowledge. In addition, the participants liked the idea of simulation that helped them predict what they would see in real-life. Furthermore, the encounters helped them communicate confidently. These findings were similar to those of some previously published studies [16, 35]. For instance, one study using pharmacy students found that the use of SPs resulted in better performance of the students during examinations [16]. Another study found that the use of SPs during an OSCE gave pharmacy students more confidence and a better ability to communicate [35]. Similarly, a study that examined nursing students’ perceptions of SP use in health assessments found that the students rated the use of SPs highly [36].

In exploring the qualitative data, more details about students’ preferences emerged and these findings intersect and corroborate with the quantitative findings discussed above. The majority of the participants preferred having SPs to faculty members playing the role of patients. This finding was consistent with the quantitative phase results and with previous studies [8, 32]. For instance, a previously published study found that pharmacy students preferred interactions with community volunteers over staff members and their peers [32]. In another study, most pharmacy students reported that the interaction with SPs was positive and helpful [8]. Another point raised by the participants during the FGDs was the simulation of real life. Most of the participants found that the interactions with SPs simulated real life. This was also previously reported by some studies, such as a qualitative study by Koo et al. in which they found that utilization of SPs made the cases better simulate real life according to the participants in the study [37]. Some of the participants raised the concern of having some incompetent SPs who make interactions extremely difficult during assessments (FG4, AS). This is a valid challenge that could be faced during the utilization of SPs in education and was reported by some previous studies [38, 39].

The participants in the FGDs provided essential recommendations to improve the SP program. Many of these recommendations were highlighted by previous studies. For example, some participants raised the point about providing the SPs with examples of ideal interactions. This finding was also reported in a previous review article that found that some schools make SPs watch videos with examples of ideal interactions in an attempt to improve performance [39]. SPs providing feedback to students was recommended by some participants of this study to improve the interactions between the SPs and students. This was also reported previously as a substantially important step to improve students’ performance and satisfaction with SP programs [28].

The major strength of this study was the use of a rigorous mixed-methods design. It is believed that mixed-methods research has the potential to generate quality research evidence by combining strengths and overcoming the respective limitations of qualitative and quantitative methodologies [25, 30, 40]. Another strength of this study is the adequate representation of the study population as we have participants who met the inclusion criteria from different pharmacy student batches. The quantitative part of the study has a limitation of the small sample size, which can affect the generalizability of the findings. However, QU-CPH is the only pharmacy school in Qatar with an average number of students per professional year ranging between 25 and 35 students. Therefore, the whole population is naturally small. Overall, the response rate was high for this study, exceeding 77%. Another potential limitation of this study is the potential for social desirability bias as the study investigators were their faculty members. However, social desirability bias was not noticed during the qualitative phase of the study.

## Conclusion and lessons learned

This study showed a positive impact of the utilization of SPs in teaching and learning and the assessment of professional skills and patient assessment courses in this pharmacy curriculum as perceived by students and alumni. Moreover, it was generally perceived that SPs add multiple values in terms of simulation-based education and the preparation of the students for their professional careers. However, the SP program needs to be optimized in terms of the training and orientation of SPs to avoid potential deficiencies in the standardization of cases and simulation of real life. We recommend expanding this study in the future to include all health professional education colleges that utilize SPs in the university to obtain more comprehensive knowledge on the status of SP utilization and develop action plans to optimize this vital program.

## Data Availability

The raw data and anonymized/pseudonymized data are available on request from the corresponding author, if required. The data are not publicly available due to ethical restrictions.

## Abbreviations

SPs: Simulated patients
QU: Qatar University
CPH: College of Pharmacy
OSCE: Objective Structured Clinical Examination
SMSA: Structured Multi Skill Assessment
FGDs: Focus group discussions
FG: Focus groups
SPSS: Statistical Package for Social Sciences
SRQR: Standards for Reporting Qualitative Research
